# Assessing the Antioxidant Properties, In Vitro Cytotoxicity and Antitumoral Effects of Polyphenol-Rich *Perilla leaves* Extracts

**DOI:** 10.3390/antiox13010058

**Published:** 2023-12-29

**Authors:** Gigi Adam, Florina Daniela Cojocaru, Liliana Verestiuc, Oana Cioanca, Ingrid-Andrada Vasilache, Ana-Maria Adam, Cornelia Mircea, Aurel Nechita, Valeriu Harabor, Bogdan Huzum, AnaMaria Harabor, Monica Hancianu

**Affiliations:** 1 Faculty of Pharmacy, “Grigore T. Popa” University of Medicine and Pharmacy, 16 Universitatii Street, 700115 Iasi, Romaniacornelia.mircea@umfiasi.ro (C.M.); monica.hancianu@umfiasi.ro (M.H.); 2 Faculty of Medicine and Pharmacy, “Dunarea de Jos” University, 35 Al. I. Cuza Street, 800216 Galati, Romania; aurel.nechita@ugal.ro (A.N.); valeriu.harabor@ugal.ro (V.H.); ana.harabor@ugal.ro (A.H.); 3 Department of Biomedical Sciences, Faculty of Medical Bioengineering, University of Medicine and Pharmacy “Grigore T. Popa” Iasi, 700454 Iasi, Romania; florina.cojocaru@umfiasi.ro (F.D.C.); liliana.verestiuc@umfiasi.ro (L.V.); 4 Department of Obstetrics and Gynecology, “Grigore T. Popa” University of Medicine and Pharmacy, 700115 Iasi, Romania; 5 Department of Orthopaedic and Traumatology, Faculty of Medicine, “Grigore T. Popa” University of Medicine and Pharmacy, 16 Universitatii Street, 700115 Iasi, Romania

**Keywords:** *Perilla frutescens*, extracts, antioxidant, antitumoral, cytotoxicity

## Abstract

(1) Background: This study aimed to outline the antioxidant, antitumoral, and cytotoxic proprieties of various types of *Perilla frutescens* extracts obtained from the leaves of the species. (2) Methods: We determined total polyphenols, flavonoids and anthocyanins contents, as well as the in vitro antioxidant, antitumoral, and cytotoxic actions in three types of ethanolic extracts (E1, E2, E3) and in three types of acetone: ethanol extracts (A1, A2, A3) of *Perilla frutescens* according to standardized procedures. (3) Results: We found that *Perilla frutescens* ethanolic extracts had the highest total phenol and anthocyanins concentrations. The flavonoids concentration was not statistically different between the extracts. The iron chelating capacity, hydroxyl radical scavenging capacity, superoxide anion radical scavenging capacity, and lipoxygenase inhibition capacity showed a significant increase with higher concentrations of *Perilla frutescens* extracts, particularly the ethanolic extracts. Perillyl alcohol had greater cytotoxic capacity in the MG-63 cell line and E1 extract showed similar significant cytotoxic effects in the A431 cell line. (4) Conclusions: Both ethanolic and acetone–ethanol extracts from *Perilla frutescens* exhibited important antioxidant and antitumoral actions in vitro, which proportionally increased with concentration. The cytotoxic threshold determined in this study for various types of extracts could help determine the best dosage with the maximum antioxidant and antitumoral potential. Our results could serve as a basis for further studies that will investigate the cytotoxic effects of *Perilla frutescens* variants on various types of cancer cell lines.

## 1. Introduction

*Perilla frutescens* (L.) Britton var. *frutescens* is a medicinal herb that belongs to the *Lamiaceae* family, with important antioxidant and antitumoral effects demonstrated both in vitro and in vivo [1,2]. Numerous types of extracts and components have been identified from this plant to date, each comprising a specific portfolio of biochemical activities and cytotoxic effects.

A recent systematic review outlined 14 classes of *Perilla frutescens* active constituents: alkaloids phenylpropane, terpenoids, polyphenol compounds, flavonoids, anthocyanins, coumarins, carotenoids, neolignans, fatty acids, tocopherols, phytosterols, glucosides and peptides [3]. These classes reunite a plethora of compounds that have antitumoral effects by modulating various elements of the metabolic pathways or intercellular interactions such as reactive oxidative species (ROS), nuclear factor kappa-light-chain-enhancer of activated B cells (NF-κB), phosphoinositide 3-kinase/protein kinase B (PI3K/AKT), c-Jun N-terminal kinases (JNK), yes-associated protein/WW domain-containing transcription factor (YAP/TAZ), etc. [3,4].

Further studies outlined the antitumoral and cytotoxic properties of phenolic and flavonoid compounds, which are abundant in water and ethanol extracts of *Perilla frutescens* [5,6]. A recent study by Garcia et al. investigated the in vitro cytotoxic effects of various parts of *Perilla frutescens* extracts (stems, leaves, and seeds) on prostate cancer cells (DU-145) [7]. The authors demonstrated that the leaf extracts possessed the highest amounts of phytochemicals compared to the other studied parts, and that the ethanolic extract from the leaves exhibited the highest cytotoxic potential on DU-145 cells.

The antioxidant proprieties of *Perilla frutescens* extracts were demonstrated in numerous trials and have an important impact throughout the evolution of metabolic or degenerative disorders [8,9,10]. The metabolite profiles of *Perilla frutescens* extracts vary depending on the species, the part of the plant used, the type of extract, and the method of determination, providing numerous possibilities to study their antioxidant proprieties.

During inflammatory processes, a large amount of superoxide radicals, along with other radicals, are produced by activated neutrophils and macrophages under the action of NAD(P)H oxidase. These free radicals generated in the inflammatory focus can induce local and general toxic phenomena. Anti-inflammatory drugs reduce the synthesis of pro-inflammatory compounds, but generally do not have the ability to neutralize the pro-oxidant compounds generated during the inflammatory process. Thus, compounds with antioxidant action can represent an important therapeutic option [11,12,13].

Polyphenols, effective antioxidants identified from *Perilla frutescens* extracts, are secondary metabolites that have the ability to neutralize hydroxyl and superoxide radicals, to block the oxidizing action of peroxynitrite that affects the structure of biologically active molecules and to chelate pro-oxidizing transitional metals. The antioxidant action is complemented by the anti-inflammatory, antibacterial, enzyme inhibitory and antimutagenic [14].

Flavonoids act as scavengers of free radicals due to their ability to donate hydrogen atoms to radicals and stabilize them. The scavenger capacity of flavonoids depends on their structure, on the number of hydroxyl groups in the structure, on the position of these hydroxyl groups, and on the ability of the hydroxyl groups to give up hydrogen atoms [15].

The scavenging capacity of free radicals is conditioned by the presence of hydroxy groups in the 3′, 4′ positions of the B ring in the flavonoid structure. The hydroxy groups in the 3′, 4′ positions of the B ring also influence the ability of flavones to fix transition metals. Methoxylation of hydroxy groups causes the disappearance of the scavenger action [16].

An important role is also played by the OH group in position 3 of ring C. The C2–C3 double bond conjugated with a keto group in position 4 determines the delocalization of electrons in the B nucleus and increases the scavenger capacity of free radicals, while the reduction of this bond will decrease the scavenger potential of the flavone without the antiradical action disappearing [15].

The presence of the hydroxyl group in positions 3 (nucleus A) and 5 (nucleus C) and a carbonyl group in position 4 (nucleus C) increases the scavenger capacity. In the absence of o-dihydroxy groups in the structure of the B nucleus, the hydroxy groups in the A nucleus are capable of inducing the scavenger action of free radicals [15]. The position of hydroxyl groups in the structure of flavonoids is more important compared to their number.

The most active flavonoids are those that have hydroxyl groups in positions 3 and 4 of nucleus B and/or hydroxyl groups in position 3 of nucleus C. Hydroxyl groups in nucleus B also increase the stability of flavonoids. Flavones that do not have OH groups in the B nucleus, but have an OH group in position 3 of the C nucleus and a keto group in position 4, have the scavenger action [10].

Glycosylation at the carbon atom increases the antioxidant character, and glycosylation at the oxygen atom decreases the antioxidant capacity [14].

Flavones can inhibit enzymes that catalyze the synthesis of reactive oxygen species, such as NADH oxidase, mitochondrial succinoxidase and microsomal monooxygenase. They also increase the activity of antioxidant enzymes: superoxide dismutase, catalase, glutathione peroxidase and glutathione reductase [17,18].

The cytotoxic effects of different varieties of *Perilla frutescens* and types of extracts from these varieties are not extensively studied in the literature. Thus, in this study, we aimed to characterize three varieties of *Perilla frutescens* species, namely *Perilla frutescens* var. *crispa* f. *purpurea*, *Perilla frutescens* var. *frutescens* f. *crispidiscolor*, and *Perilla frutescens* var. *frutescens* f. *viridis*, and their corresponding extracts (ethanolic or ethanol–acetone) considering their chemical composition, as well as their in vitro antioxidant, cytotoxic and antitumoral proprieties. 

This study will comprise three parts corresponding to our primary objectives: (a) quantification of the total flavonoid, polyphenols, and anthocyanin content of various types of *Perilla frutescens* extracts; (b) determination of the in vitro antioxidant action of *Perilla frutescens* extracts; (c) characterization of the in vitro cytotoxic and antitumoral activity of *Perilla frutescens* extracts.

## 2. Materials and Methods

### 2.1. The Extraction Processes

The study focused on the chemical analysis of two types of extracts obtained by processing the plant material harvested from three varieties of *Perilla frutescens* var. *crispa* f. *purpurea*, *Perilla frutescens* var. *frutescens* f. *crispidiscolor*, and *Perilla frutescens* var. *frutescens* f. *viridis*. The plant material consisted of leaves harvested in September 2020 at the Vegetable Research and Development Station in Buzău, Romania, with the following coordinates: 45°09′30.2″ northern latitude and 26°49′37.9″ eastern longitude. Cultivation was carried out both in greenhouses and in the open field between May and September 2020. Taxonomic identification was provided by a specialized biologist, and the vegetal material is currently deposited in the collection of conserved materials from the Vegetal Morphoanatomy Laboratory at Biology Faculty of the “Alexandru Ioan Cuza” University, Iasi, Romania.

The harvested leaves were dried to a constant weight over a period of three weeks. They were placed on white paper sheets in a thin layer in a dark and well-ventilated room at a temperature of 23 °C. The plant material was used to obtain two types of extracts, which were employed for both qualitative and quantitative chemical characterization of polyphenolic compounds.

For the first type of extract, 2 g of pulverized plant material was placed in round-bottom flasks and 50 mL of 70% ethanol was added. The mixture was subjected to reflux extraction at a temperature of 70 °C using a thermostatic water bath for a duration of 6 h, a process repeated twice. The extraction products were filtered and then combined.

In the case of the second type of extract, 2 g of pulverized plant material was placed in iodometric flasks, and 100 mL of a solvent mixture of acetone–ethanol (7:3) and 0.5 g of citric acid was added. Each flask was equipped with a magnetic stir bar, tightly sealed, and subjected to extraction on a magnetic stirrer for 6 h at room temperature. This process was repeated twice. Following extraction, the obtained filtrates were combined.

The extracts obtained after combining the filtrates were transferred into porcelain capsules and left at room temperature for concentration and drying. The resulting extracts were utilized for both chemical and biological characterization.

We choose to study these types of extracts because ethanol is known for its high extraction efficiency and its ability to extract bioactive compounds from various plant materials. It is a common choice for extracting phytochemicals, such as phenolic compounds, flavonoids, and alkaloids. Also, acetone–ethanol mixtures can provide a balance between polar and non-polar extraction, allowing for a more comprehensive extraction of a diverse array of compounds.

### 2.2. Total Flavonoids, Phenols, and Anthocyanins Quantification 

#### 2.2.1. Total Flavonoids Quantification

Flavonoids were quantified as previously described, and their concentration was expressed as rutoside equivalents (µg/mL). Briefly, 5 mL of each extract sample was mixed with 5.0 mL of 100 g/L sodium acetate and 3.0 mL of 25 g/L aluminum chloride. Methanol was added up to 25 mL in a graduated flask. The absorbance corresponding to the yellow color of the complex was photometered at 430 nm (the corresponding wavelength for optimal absorption for rutoside). The final results represent the average of three determinations and the standard deviation.

#### 2.2.2. Total Phenol Quantification

Total polyphenols were quantified according to the well-established methodology using gallic acid as standard. For this, 40 microliters of the solution of each extract were mixed with 3160 microliters of distilled water and 200 microliters of Folin–Ciocâlteu reagent. After 5 min, 600 microliters of 20% sodium carbonate solution was added. After 2 h for incubation in the dark, the absorbance of the mixture was read at λ = 716 nm against a compensation liquid consisting of 40 μL extract solution, 3760 μL distilled water and 200 μL Folin–Ciocâlteu reagent. This technique indicates the presence of free hydroxyl groups that are available in all compounds found in the investigated sample. The final results expressed as µg gallic acid/mL extract represent the average of three determinations ± standard deviation. 

Calibration curves in rutoside (concentration ranges 0.025–0.3 mg/mL, R^2^ = 0.9906) and gallic acid (concentration ranges 0.039–2.5 mg/mL, R^2^ = 0.9969) were established in parallel for each group of components, using the same methodology.

#### 2.2.3. Total Anthocyanin Quantification

Starting from the method described by Giusti and Wrolstad in 2001, the total anthocyanin content was evaluated by using two dilutions of the same extract and changing the pH between 1.0 and 4.5. It is known that the chemical form of anthocyanin radicals (oxonium) has different absorption spectra when the acidic environment changes [19]. 

A quota from each extract was diluted to 10 mL in two volumetric flasks, adjusting the pH to 1.0 (potassium chloride buffer), and the other to pH 4.5 (sodium acetate buffer), mixing continuously. After an equilibration time (15–20 min), the absorbance of each dilution was measured at 510 nm and then 700 nm against a blank of distilled water. The difference between each measurement at both pH values represents the corrected absorbance used for further calculation. The results were expressed as µg cyanidol/mL extract and were calculated as the average of three determinations.

#### 2.2.4. UHPLC-PDA Quantification and Identification of Extracted Compounds

In the present research, a Thermo Fischer UltiMate 3000 ultra-fast system (Thermo Fisher Scientific Inc., Waltham, MA, USA) coupled with a multi-diode detector (PDA), quaternary pump (LPG-3400 SD) with built-in four-channel degasser and an autosampler (injecting variable amounts from 2 μL to 200 μL) from the same producer, was used. 

The conditions for UHPLC-PDA included Kinetex C18 (150 × 4.6 mm, 100 Å) column (Phenomenex, Torrance, CA, USA), and a gradient of 10 to 65 A (acetonitrile) in B (aqueous solution of acetic acid 0.1%) over 25 min, a starting flow of 1 mL/min, then 0.8 mL/min.

Each sample was obtained from 4 mg of dry extract diluted to 1 mL (ethanol–water, 3:1), from which 10 µL were injected for analysis. Simultaneous detection at three wavelengths 275 nm (flavonoids), 330 nm (polyphenolic acids), 520 nm (anthocyanins) was used. 

The integration, identification and calculation of each peak was carried out with Chromeleon™ 7.3 (Thermo Scientific™ Dionex™, Thermo Fisher Scientific Inc., Waltham, MA, USA). A matching index of at least 950 (from a total of 1000) was used to identify compounds based on UV spectra and retention time.

Aliquots (2–10 µL) of standard stock solutions (epicatechin, caffeic acid, rosmarinic acid, chlorogenic acid, ferulic acid, luteolin, apigenin, quercetin-3-arabinoside, apigenin-7-O-glucoside, and luteolin 7-O-glucoside) were used for calibration curves with a correlation coefficient above 0.9989. The standard deviation was calculated at 0.02. The limit of detection (LOD) and the limit of quantification (LOQ) of rutoside and rosmarinic acid were calculated at 272 ng/mL and 182 ng/mL, respectively. 

#### 2.2.5. Chemicals and Materials

The reagents used for spectrophotometric determinations (sodium acetate, aluminum chloride, Folin–Ciocalteu phenol reagent, reagent-grade sodium carbonate, sodium acetate buffer, potassium chloride buffer) and solvents were bought from Merck (Darmstadt, Germany)

All standards and solvents were of HPLC (Chromasolv) quality and were purchased either from Sigma-Aldrich or from Merck (Darmstadt, Germany). The pure compounds used as standards were obtained from Sigma-Aldrich (Darmstadt, Germany) and the purity grade was at least 95%.

### 2.3. Iron Chelation Capacity Assessment

Principle of the Method: Fe^2+^ forms a pink-colored complex with ferrozine, exhibiting maximum absorbance at 562 nm. The presence of a chelating agent in the reaction medium results in reduced absorbance of the formed complex, leading to a decrease in the solution’s color intensity [20,21]. The list of the reagents used for this procedure included:

- Dimethyl sulfoxide, DMSO (Merck, KgaA, Darmstadt, Germany);
- 0.1 M Acetate Buffer, pH 5.25;
- Iron (II) sulfate heptahydrate (Merck, KgaA, Darmstadt, Germany);
- 2 mM Iron (II) sulfate in 0.2 M HCl;
- Ferrozine (sodium salt of 3-(2-pyridyl)-5,6-diphenyl-1,2,4-triazine-4′,4′’ disulfonic acid) (Fluka, Sigma-Aldrich, Steinheim, Germany);
- 5 mM Ferrozine Solution;
- Hydrochloric Acid (Merck, Darmstadt, Germany);
- UV-VIS Spectrophotometer ABL & E Jasco V-550 (Tokyo, Japan);
- pH Meter Hanna Instruments pH210, Microprocessor pH-meter;
- Vortex Mixer Velp Scientifica (Usmate Velate, Italy);
- Ultra Clear TWF Water Purification System;
- Test samples—solutions obtained by dissolving dried extracts in DMSO—hydroalcoholic extracts (E1, E2, E3), extracts in acetone: ethanol (7:3) acidified (A1, A2, A3); solution concentration for analysis: 0.078125–10 mg/mL.

The procedure consisted of the following steps:

- We mixed 0.2 mL of the test sample solution in ultrapure water, 0.74 mL of 0.1 M acetate buffer (pH 5.25), and 0.02 mL of 2 mM iron sulfate in 0.2 M hydrochloric acid. After 10–15 s of agitation, we added 0.04 mL of 5 mM ferrozine solution.
- After 10 min of incubation in the dark, we measured the absorbance of the solution at 562 nm against a control prepared under the same conditions as the sample (ultrapure water was used instead of iron sulfate solution).
- Simultaneously, we prepared the control solution and its control: the control contained 0.2 mL of ultrapure water, 0.74 mL of 0.1 M acetate buffer (pH 5.25), 0.02 mL of 2 mM iron sulfate in 0.2 M hydrochloric acid, and 0.04 mL of 5 mM ferrozine solution.
- Gallic acid was used as a reference substance, and gallic acid solutions in DMSO were processed under the same conditions as the ethanolic extract.
- All determinations were carried out in triplicate, and results were expressed as the mean of three determinations ± standard deviation.

The chelating capacity of the ferrous ion was calculated using the Formula (1):

% Activity = 100 × (Ac − Ap)/(Ac)(1)

where:

Ac is the absorbance of the control solution.

Ap is the absorbance of the sample solution.

For samples exhibiting a ferrous ion chelation capacity of over 50%, the CE50 value was calculated and expressed in mg of sample/mL of final solution. CE50 was calculated by considering the first value below 50% and the first value above 50% and interpolating linearly to determine the concentration of the antioxidant agent corresponding to 50% activity.

### 2.4. Determination of Hydroxyl Radical Scavenging Capacity

Principle of the Method: The hydroxyl radical, formed in the reaction between ferrous ion and hydrogen peroxide, will hydroxylate salicylic acid, resulting in the formation of a pink–violet compound with maximum absorbance at 562 nm [22]. A list of the reagents used for this procedure included:

- Dimethyl sulfoxide (Merck, KgaA, Darmstadt, Germany);
- Ferrous sulfate heptahydrate (Merck, KgaA, Darmstadt, Germany);
- 1.5 mM ferrous sulfate solution in distilled water;
- 6 mM hydrogen peroxide solution in distilled water (Sigma-Aldrich, Steinheim, Germany);
- 20 mM sodium salicylate solution in distilled water;
- Hanna Instruments pH210 Microprocessor pH-meter (Padova, Italy);
- Velp Scientifica Vortex agitator (Usmate Velate, Italy);
- Ultra Clear TWF water purification apparatus (Günzburg, Germany);
- ABL and E Jasco V-550 UV-VIS spectrophotometer (Tokyo, Japan);
- Test samples—solutions obtained by dissolving dry extracts in DMSO—hydroalcoholic extracts (E1, E2, E3), extracts in acetone: ethanol 7:3 acidic mixture (A1, A2, A3); concentration of analyzed solutions: 0.078125–10 mg/mL.

Over 0.225 mL of the sample solution in dimethyl sulfoxide, 0.750 mL of 1.5 mM ferrous sulfate solution, 0.9 mL of 20 mM sodium salicylate solution, and 0.525 mL of 6 mM hydrogen peroxide solution were added. 

The mixture was kept at 37 °C for 30 min, and after cooling to room temperature, the absorbance of the sample (control) was read at 562 nm against the sample’s (control’s) blank, in which the ferrous sulfate solution was replaced with distilled water. The positive control was processed under the same conditions as the samples, but dimethyl sulfoxide was used in place of the sample solution.

Gallic acid was used as a reference substance, and gallic acid solutions in DMSO were processed under the same conditions as the ethanolic extract. All determinations were performed in triplicate, and the results are expressed as the mean of three determinations ± standard deviation.

The hydroxyl radical scavenging capacity was calculated according to the Formula (1).

For the samples that exhibited a hydroxyl radical scavenging capacity of over 50%, the CE50 value was calculated and expressed in µg of sample/mL of the final solution or µg of gallic acid/mL. CE50 was calculated by considering the first value lower than 50% and the first value higher than 50%, obtaining, through linear interpolation, the concentration of the antioxidant solution that corresponds to a 50% activity.

### 2.5. Determining the Scavenging Capacity of the Superoxide Radical Anion

Principle of the Method: The superoxide radical generated by the reduced nicotinamide adenine dinucleotide-phenazine methosulfate system reduces nitroblue tetrazolium to a violet-blue formazan compound with maximum absorbance at 560 nm [23]. 

The following reagents were used:

- Dimethyl sulfoxide (Merck, KgaA, Darmstadt, Germany);
- TRIS (Sigma-Aldrich, Steinheim, Germany);
- TRIS buffer pH 8—0.4845 g of TRIS dissolved in 180 mL of distilled water, adjusted to pH 8 with 6M HCl solution, and completed with distilled water to 200 mL;
- Reduced nicotinamide adenine dinucleotide sodium salt (NADHNa2) (Sigma-Aldrich, Steinheim, Germany);
- 557 µM solution of reduced nicotinamide adenine dinucleotide sodium salt (NADHNa2) in TRIS buffer pH 8;
- nitroblue tetrazolium (Fluka, Steinheim, Germany);
- 108 µM solution of nitroblue tetrazolium in TRIS buffer pH 8;
- Phenazine methosulfate (Fluka, Sigma-Aldrich, Steinheim, Germany);
- 45 µM solution of phenazine methosulfate in TRIS buffer pH 8;
- pH meter Hanna Instruments pH210, Microprocessor pH-meter (Padova, Italy);
- Vortex mixer Velp Scientifica (Usmate Velate, Italy);
- Ultra Clear TWF water purification apparatus (Günzburg, Germany);
- UV-VIS spectrophotometer ABL & E Jasco V-550 (Tokyo, Japan);
- Test samples—solutions obtained by dissolving dried extracts in DMSO—hydroalcoholic extracts (E1, E2, E3), extracts in a mixed solution of acetone: ethanol 7:3 (A1, A2, A3); concentration of the analyzed solutions: 0.078125–10 mg/mL.

Over 0.5 mL of sample solution in dimethyl sulfoxide (diluted solutions in dimethyl sulfoxide) 0.5 mL of 557 µM NADHNa2 solution in TRIS buffer pH 8, and 0.5 mL of 108 µM nitroblue tetrazolium solution in TRIS buffer pH 8 were added. The mixture was vortexed for 5 s. To the mixture, 0.5 mL of 45 µM phenazine methosulfate solution in TRIS buffer pH 8 was added, and it was allowed to stand for 5 min at room temperature. Afterward, the absorbance of the sample (control) was measured against the sample control at 560 nm. The positive control was processed under the same conditions as the samples, but dimethyl sulfoxide was used instead of the sample solution.

Gallic acid was used as a reference substance, and solutions of gallic acid in DMSO were processed under the same conditions as the ethanolic extract. All determinations were performed in triplicate, and the results were expressed as the mean of three determinations ± standard deviation.

The hydroxyl radical scavenging capacity was calculated according to the Formula (1).

For the samples that exhibited a superoxide radical scavenging capacity of over 50%, the CE50 value was calculated and expressed in µg sample/mL final solution or µg gallic acid/mL. The CE50 was calculated by considering the first value lower than 50% and the first value higher than 50%, and then obtaining, through linear interpolation, the concentration of the antioxidant solution corresponding to a 50% activity.

### 2.6. Determination of the Lipoxygenase Inhibition Capacity of Perilla frutescens Extracts

Principle of the Method: The active compounds present in the extracts block 15-lipoxygenase by inhibiting the oxidation of linoleic acid and reducing absorbance at 234 nm [24]. 

The reagents used included:

- Dimethyl sulfoxide (DMSO) (Merck, KgaA, Darmstadt, Germany);
- 0.1 M borate buffer pH 9—Dissolve 6.2 g of boric acid in 950 mL of distilled water, adjust to pH 9 with 1M NaOH, and make up to 1000 mL with distilled water;
- Linoleic acid (Sigma-Aldrich, Steinheim, Germany) 0.16 mM in 0.1 M borate buffer pH 9;
- Soybean lipoxygenase (Sigma-Aldrich, Steinheim, Germany) in 0.1 M borate buffer pH 9;
- UV-VIS spectrophotometer ABL & E Jasco V-550 (Tokyo, Japan);
- pH meter Hanna Instruments pH210, Microprocessor pH-meter (Padova, Italy);
- Vortex mixer Velp Scientifica (Usmate Velate, Italy);
- Ultra Clear TWF water purification system (Günzburg, Germany);
- Test samples—solutions obtained by dissolving dried extracts in DMSO—hydroalcoholic extracts (E1, E2, E3), extracts in a mixture of acetone: ethanol 7:3 (A1, A2, A3); concentration of the analyzed solutions: 0.078125–10 mg/mL.

Firstly, 0.05 mL of 15-lipoxygenase solution in borate buffer pH 9 was treated with 0.05 mL of the diluted test solution in DMSO, and the mixture was left to stand for 10 min at room temperature. Afterward, 2 mL of 0.16 mM linoleic acid solution in 0.1 M borate buffer pH 9 was added. The absorbance of the solution was recorded at 234 nm within the 0–120 s interval. In parallel, a positive control was processed in which the test solution was replaced with DMSO.

Gallic acid was used as a reference substance, and solutions of gallic acid in DMSO were processed under the same conditions as the ethanolic extract. All determinations were carried out in triplicate, and the results were expressed as the mean of three determinations ± standard deviation.

The lipoxygenase inhibition capacity was calculated using the formula:

% Activity = (AEFI − AECI) × 100/AEFI(2)

where:

AEFI—represents the difference between the absorbance of the enzyme solution without an inhibitor at 90 s and the absorbance of the same solution at 30 s; 

AECI—represents the difference between the absorbance of the enzyme solution treated with an inhibitor (test sample or gallic acid) at 90 s and the absorbance of the same solution at 30 s.

For samples that exhibited a lipoxygenase inhibition capacity of more than 50%, the CE50 value was calculated and expressed in µg of sample/mL of the final solution or µg of gallic acid/mL. CE50 was calculated by taking into account the first value below 50% and the first value above 50%, obtaining, through linear interpolation, the concentration of the antioxidant solution corresponding to 50% activity.

### 2.7. In Vitro Cytotoxicity Tests and Antitumor Action of Perilla Leaves Extracts

For the study of material cytotoxicity, two human tumor cell lines were used: human osteosarcoma cells (MG-63 cell line from ATCC, Rockville, MD, USA) and tumor keratinocytes (A431 cell line from Cell Service, Eppelheim, Germany). The cells were separately incubated for 24 h (5% CO_2_, 37 °C, 95% relative humidity) in DMEM culture medium enriched with 10% FBS and 1% P/S/N, in 96-well plates for the MTT assay (2 × 10^3^ cells/well for MG-63 and 3 × 10^3^ cells/well for A431) and in 48-well plates for cell morphology studies (8 × 10^3^ cells/well for MG-63 and 1 × 10^4^ cells/well for A431). After 24 h, the medium in the plates was replaced with fresh DMEM medium (with 10% FBS and 1% P/S/N) for the control, and with extracts prepared according to the protocol described in the following paragraph.

For the in vitro cytotoxicity evaluation, an indirect contact method was used. A certain amount (2 mg/mL stock solution) of each material was immersed in DMEM and 1% P/S/N and left to shake (200 rpm, 37 °C) for 24 h. Afterward, the stock solution was obtained by passing the medium through 0.22 μm filters, and finally, 10% FBS was added. For each material, the MTT test was performed for 6 different extract concentrations: 2 mg/mL, 1 mg/mL, 0.5 mg/mL, 0.2 mg/mL, 0.1 mg/mL, and 0.05 mg/mL.

For the MTT assay, the culture medium and extracts from the wells were replaced with MTT working solution (5% MTT in DMEM without FBS and P/S/N). The culture plates were incubated at 37 °C for 2 h, during which viable cells reduced the tetrazolium salt to a colored product called formazan, solubilized with DMSO. 

The absorbance of the resulting formazan solution (blue–violet color) was measured spectrophotometrically at λ = 570 nm using a plate reader (Tecan Sun-rise Plate Reader, Tecan Trading AG, Männedorf, Switzerland). The spectrofotometric readings from the experimental wells were reported relative to the control wells, where no extracts were present. The calculated ratio represented cell viability (V):

(3) $$V\;=\;\frac{abs\;extract}{abs\;control}\times 100$$

where:

abs extract—the absorbance of the extract;

abs control—the absorbance of the control.

The MTT assay was conducted at 24, 48, and 72 h, in triplicate, and analyzed by means of two-way ANOVA followed by Bonferroni’s post hoc test. A *p*-value of less than 0.05 was accepted as significant. We used the Compact Letter Display (CLD) system for coding the statistical significance in the ANOVA test as follows: (a) variables with indistinguishable means were assigned the same letter; (b) the variable with the highest mean (or average) will be named “a”.

The Calcein-AM Cell Viability Assay was conducted at 72 h of contact. In the initial stage, the culture medium in the wells was removed, and then the cells were washed twice with HBSS containing calcium and magnesium, without phenol red. 

Finally, a calcein solution was added (2 µL calcein per 1 mL HBSS with calcium and magnesium, without phenol red), and the culture plate was incubated at 37 °C, 5.5% CO_2_, and 96% relative humidity for 40 min. To study the cell morphology, an inverted fluorescence microscope (Leica Microsystems GmbH, Wetzlar, Germany) was used, and images were captured.

### 2.8. Statistical Analyses

Continuous data were compared among multiple groups using ANOVA analysis, followed by Bonferroni’s post hoc test. Student’s *t*-test was used to compare the numerical variables of two groups. A *p*-value less than 0.05 was considered statistically significant. These analyses were performed using STATA SE (version 17, 2023, StataCorp LLC, College Station, TX, USA).

## 3. Results

### 3.1. Quantification of the Total Phenols, Flavonoids and Anthocyanins from Ethanolic and Acetone: Ethanol Extracts of Perilla frutescens

Following the drying process, six extracts were obtained, consisting of two with a brown-violet color and four with a brown–green hue. These six dried extracts were coded to facilitate their handling in the research, and the extraction yield was calculated. The results are presented in Table 1. The 70% ethanol extracts exhibited the highest extraction yields compared to those obtained using the solvent mixture. 

The quantification of total phenols, flavonoids and anthocyanins in three types of ethanolic extracts (E1, E2, E3) and in three types of a mixture solvents (acetone: ethanol) extracts (A1, A2, A3) is presented in Table 2. 

Our results indicated that the total phenols and anthocyanins concentrations were significantly higher in the ethanolic extracts of *Perilla frutescens*. On the other hand, we could not find any statistical difference regarding the flavonoids concentrations between the ethanolic and acetone–ethanol extracts of *Perilla frutescens* varieties.

The results from the UHPLC analysis are presented in Table 3. Caffeic acid was significantly higher in the ethanolic extracts of *Perilla frutescens*, with the highest amount being identified in the E1 extract. Syringic acid, *p*-coumaric acid, ferulic acid, rosmarinic acid, kaempferol, isorhamnetin, and pinocembrin were also found to be significantly higher in ethanolic extracts. E1 extract had the highest amount of p-coumaric acid, isorhamnetin, and pinocembrin, while E2 extract had the highest amount of syringic acid. E3 extract was rich in ferulic acid and kaempferol. 

### 3.2. Determination of the In Vitro Antioxidant Action of Perilla frutescens Extracts

**Figure 1:**
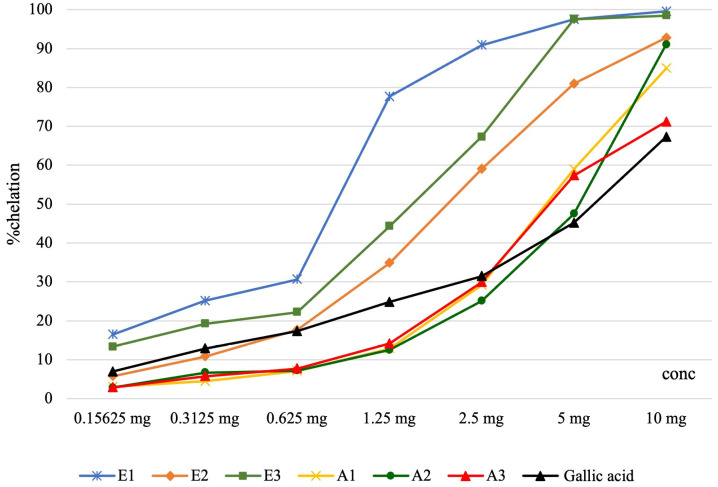
Graphical representation of the iron chelation capacity considering various concentrations of Perilla frutescens extracts.

The results from the iron chelation capacity assessment are presented in Figure 1 and Table 4. The iron chelation capacity increased with *Perilla frutescens* extracts concentrations, and it was significantly higher in ethanolic extracts compared with other types of extracts from this plant. 

**Figure 2:**
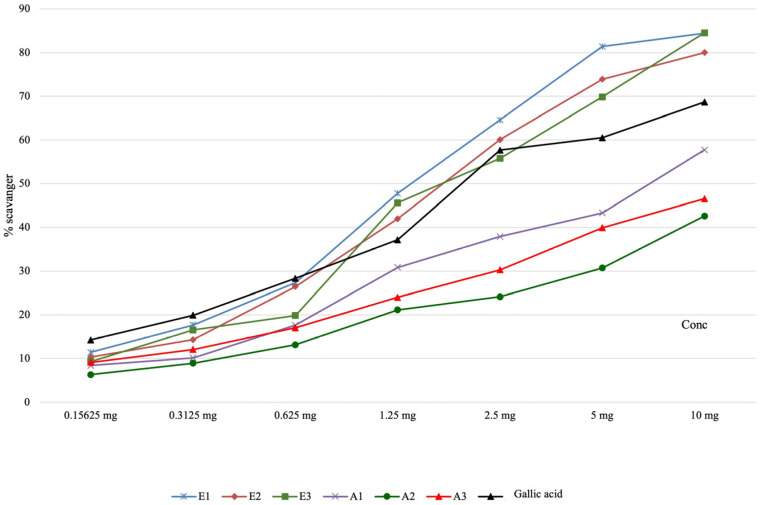
The scavenging activity of hydroxyl radical in the analyzed *Perilla frutescens* extracts.

The results obtained for the evaluation of the active principles’ capacity, present in the plant extracts, to neutralize the hydroxyl radical are presented in Table 4 and graphically represented in Figure 2. The scavenging capacity of the hydroxyl radical increased with the concentration of the analyzed *Perilla frutescens* extracts, and it was significantly higher for ethanolic extracts in comparison with other types of extracts from this plant or with gallic acid.

**Figure 3:**
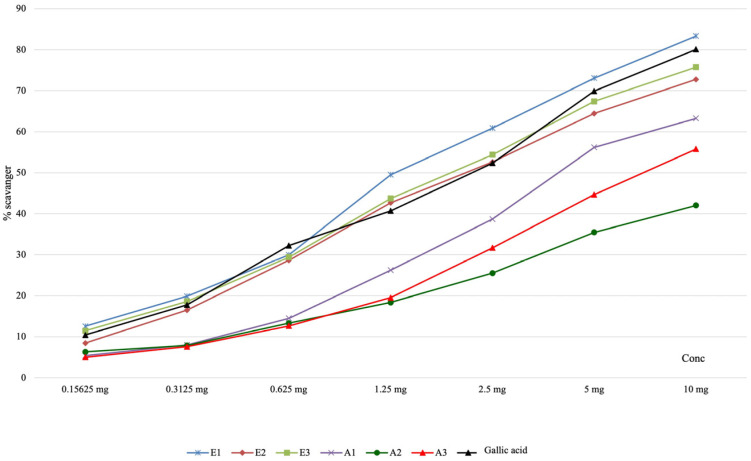
The graphical representation of the scavenging capacity of superoxide anion radicals in the analyzed *Perilla frutescens* extracts.

The results obtained from evaluating the capacity of the active principles present in the plant extracts to neutralize the superoxide anion radical are presented in Table 4 and graphically represented in Figure 3. Our results indicated that the scavenging capacity of the superoxide anion radical was significantly higher for ethanolic extracts in comparison with other types of extracts from this plant or with gallic acid.

**Figure 4:**
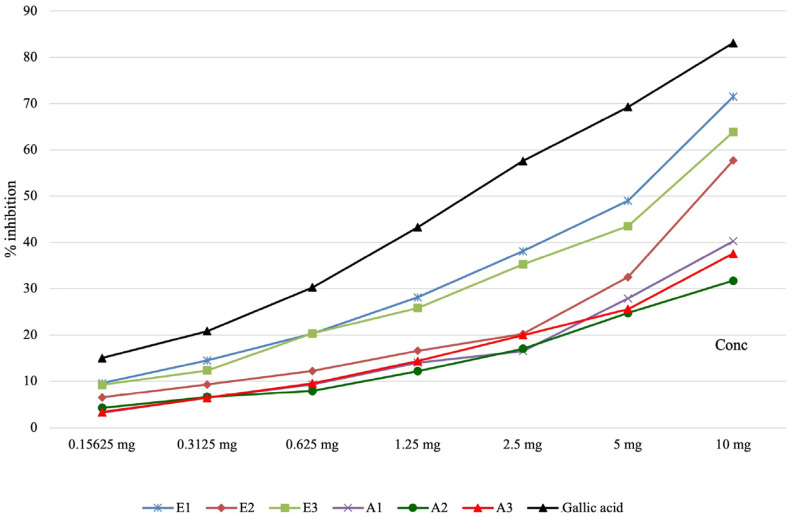
The graphical representation of the capacity of *Perilla leaves* extracts to inhibit lipoxygenase.

The results obtained from evaluating the capacity of the active principles present in plant extracts to inhibit lipoxygenase are presented in Table 4 and graphically represented in Figure 4. The lipoxygenase inhibition capacity of the analyzed *Perilla frutescens* extracts was significantly smaller compared to gallic acid. This inhibition capacity was higher for ethanolic extracts in comparison with other types of extracts from this plant.

### 3.3. Determination of In Vitro Cytotoxicity and Antitumor Effects of Perilla frutescens Extracts

**Figure 5:**
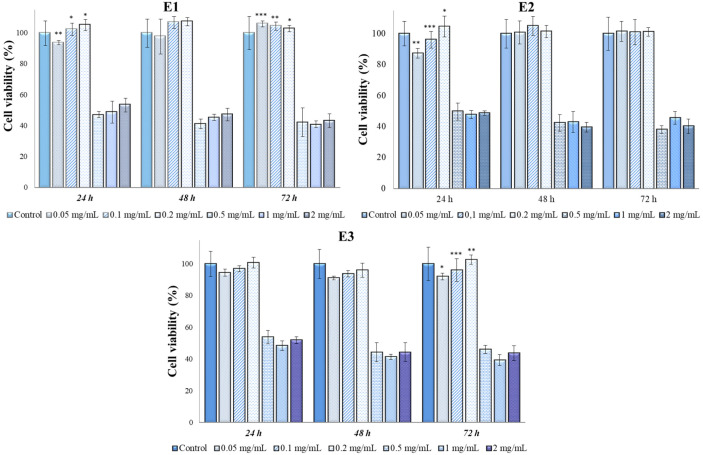
Mg-63 viability (%) in contact with E1–E3 *Perilla frutescens* extracts. Each value represents the mean ± standard error mean (n = 3). * *p* < 0.01, ** *p* < 0.001, *** *p* < 0.0001 versus control (analyzed by means of two-way ANOVA).

**Figure 6:**
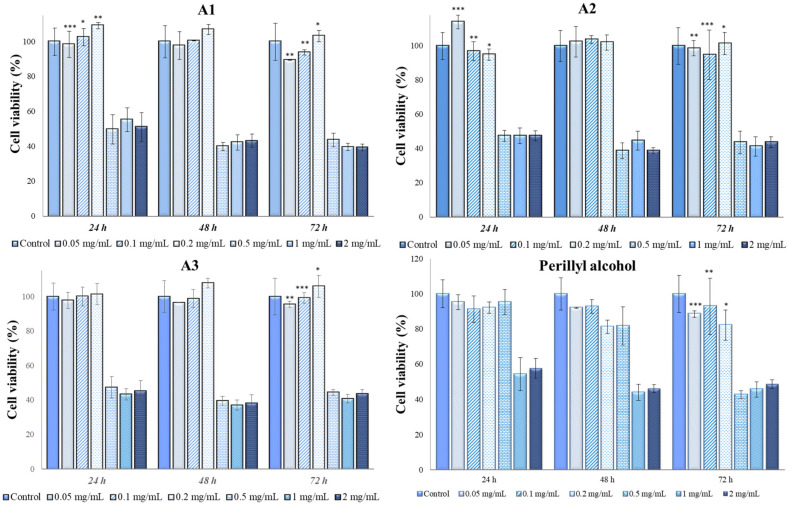
MG-63 viability (%) in contact with A1–A3 *Perilla frutescens* extracts and perillyl alcohol. Each value represents the mean ± standard error mean (n = 3). * *p* < 0.01, ** *p* < 0.001, *** *p* < 0.0001 versus control (analyzed by means of two-way ANOVA).

The results from the MTT test using various types of *Perilla frutescens* extracts and perillyl alcohol on the cellular lines MG-63 are presented in Figure 5 and Figure 6.

The antitumoral effect was observed for all types of extracts at ≥0.5 mg/mL. The cytocompatibility with the cellular lines MG-63 was observed at concentrations of 0.2 mg/mL for all types of extracts. In particular, in case of A1–A3 extracts (Figure 5), for concentrations of 0.5 mg/mL, 1 mg/mL and 2 mg/mL, the cell viability values were between 37% (48 h, A3, 1 mg/mL) and 55% (24 h, A1, 1 mg/mL), indicating a cytotoxic behavior, while for concentrations of 0.05 mg/mL, 0.1 mg/mL, and 0.2 mg/mL, the minimum values were 89% (72 h, A1, 0.05 mg/mL) and the maximum values 114% (24 h, A2, 0.05 mg/mL), indicating no toxicity. 

As can be noted in Figure 6, E1–E3 have cytotoxic effects at concentrations of 0.5 mg/mL, 1 mg/mL and 2 mg/mL, the results indicating a decrease in the viability of MG-63 cells, with values between 38% (72 h, E2) and 54% (24 h, E3). No significant differences were observed between these three concentrations, meaning that at concentrations higher than 0.5 mg/mL, extracts E1–E3 can be considered for the anti-tumor effect. In contrast, for the concentrations of 0.05 mg/mL, 0.1 mg/L and 0.2 mg/mL, the extracts did not show a cytotoxic effect, the cell viability values being between 87% (24 h, E2, 0.05 mg/mL) and 107% (48 h, E1, 0.2 mg/mL).

Perillyl alcohol showed an obvious cytotoxicity for 2 mg/mL and 1 mg/mL concentrations, while for 0.5 mg/mL and 0.2 mg/mL, close values were observed for the first two contact times (24 and 48 h), but for 72 h, in the case of the concentration of 0.5 mg/mL, a sudden decrease in the cell viability value was observed, reaching from 82% at 48 h to 43% at 72 h. For the concentrations of 0.1 mg/mL and 0.05 mg/mL, it was observed that perillyl alcohol had no cytotoxic effect in contact with MG-63 cells.

In Table 5, we present the results from ANOVA analysis with Bonferroni’s post hoc test regarding the intergroup differences of cellular viabilities in MG-63 cell line. Our analysis indicated a statistically significant difference between groups regarding the cellular viability of MG-63 cell lines when using E3 (*p* = 0.02), A1 (*p* = 0.02), and peryllyl alcohol (*p* < 0.001), the latter having the most pronounced cytotoxic effect on this type of cell line in the first 24 and 48 h. 

**Figure 7:**
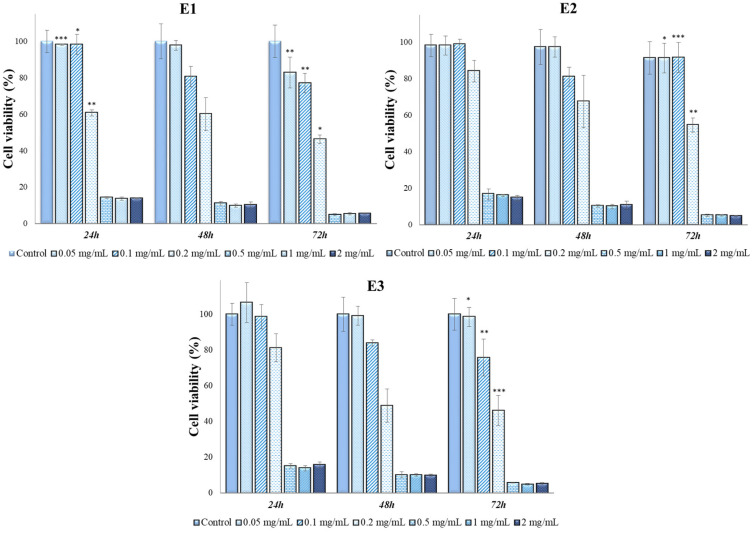
A431 viability (%) in contact with E1–E3 *Perilla frutescens* extracts. Each value represents the mean ± standard error mean (n = 3). * *p* < 0.01, ** *p* < 0.001, *** *p* < 0.0001 versus control (analyzed by means of two-way ANOVA).

**Figure 8:**
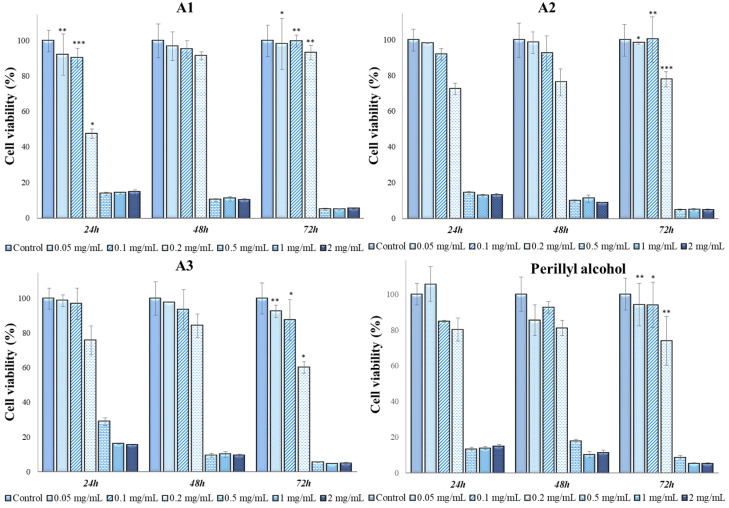
A431 viability (%) in contact with A1–A3 *Perilla frutescens* extracts and perillyl alcohol. Each value represents the mean ± standard error mean (n = 3). * *p* < 0.01, ** *p* < 0.001, *** *p* < 0.0001 versus control (analyzed by means of two-way ANOVA).

**Figure 9:**
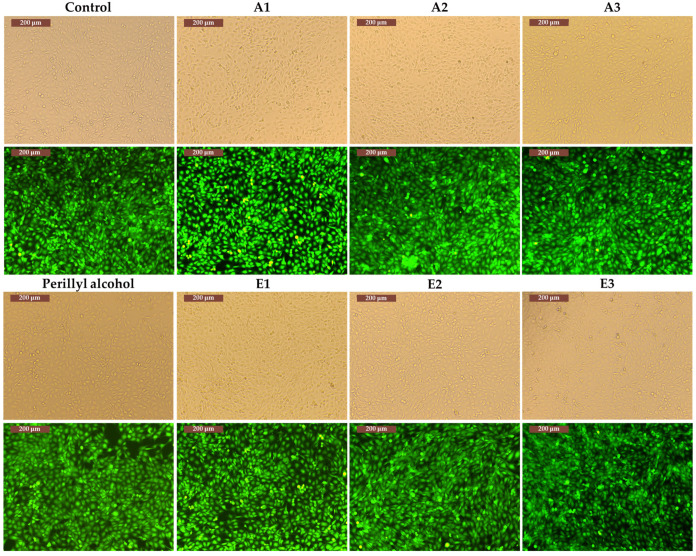
The structure and morphology of MG-63 cells cultured with extract A1–A3, E1–E3, perillyl alcohol, and the control (marked or not with Calcein-AM).

**Figure 10:**
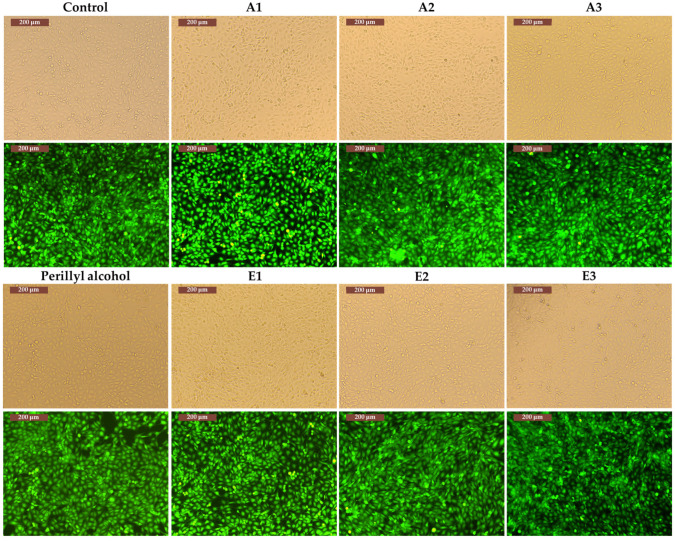
The structure and morphology of A431 cells cultured with extract E1–E3, A1–A3, perillyl alcohol, and the control (marked or not with Calcein-AM).

Since a cell line is characterized by specific mechanisms and behavior, the six extracts and perillyl alcohol were also tested in contact with the A431 cell line (tumor keratinocytes); the results are shown in Figure 7, Figure 8, Figure 9 and Figure 10.

The antitumoral effect was observed for all types of extracts at ≥0.2 mg/mL in comparison with perillyl alcohol, which exhibited an antitumoral effect at concentrations of ≥0.5 mg/mL. At 72 h of contact, cell viability values were below 9% for all analyzed samples, which indicates the possible use of *Perilla frutescens* leaf extracts with a concentration higher than 0.5 mg/mL in the treatment of skin tumors. Supplementary tests were necessary to accurately establish the dosage and mechanism of action of the extracts. 

In Table 6, we present the results from ANOVA analysis with Bonferroni’s post hoc test regarding the intergroup differences of cellular viabilities in A431 cell line. Our analysis revealed that the cytotoxic activity was similar between various concentrations of E1 (*p* = 0.43), E2 (*p* = 0.36), E3 (*p* = 0.32) and A1 (*p* = 0.16) extracts at different timeframes. On the other hand, our results indicated a statistically higher cytotoxic activity of A2 (*p* = 0.006) and A3 (*p* < 0.001) extracts in the first 24 h of contact with A431 cell line. Moreover, the cytotoxicity of perillyl alcohol was significantly higher in the 48–72 h time interval (*p* < 0.001).

As mentioned above, all six extracts, in contact with the MG-63 line, are not cytotoxic at concentrations lower than or equal to 0.2 mg/mL, but in contact with A431, it was observed that at a concentration of 0.2 mg/mL, the extracts contributed to the decrease in cell viability up to values of 46% in the case of extracts E1–E3, respectively, and 47% in the case of extracts A1–A3. At the other two analyzed concentrations, 0.1 mg/mL and 0.05 mg/mL, extracts A1, A2 and A3 had a non-cytotoxic character.

In the figures below, it can be observed that all the data obtained for the Calcein AM assay are correlated with the data obtained in the MTT test. Thus, for the MG-63 cell line, in the case of extracts E1–E3 and A1–A3, where viability values ranged from 101% to 106%, a cell density similar to that in the control wells is observed. However, in the case of perillyl alcohol, where the viability was 82%, there are areas that are not populated with cells (Figure 9).

In the case of the A431 cell line, for extracts E3 and A1–A2 (Figure 9), which showed viability values of approximately 98%, a comparable cell density can be observed compared to the control wells. For extract E1, which resulted in a 72 h cell viability, 82% (Figure 10) cell density can be observed compared to the control.

## 4. Discussion

The present study aimed to quantify the total flavonoid, phenol, and anthocyanin content, to determine the in vitro antioxidant action, and to characterize the in vitro cytotoxic and antitumoral activity of *Perilla frutescens* leaves extracts.

Our results indicated that the extraction yield was higher for ethanolic extracts compared with solvent mixture extracts, both categories had an extraction yield of more than 30%, which indicates a performance comparable to others published in the literature [25,26]. 

The total phenols and anthocyanins concentrations were significantly higher in the ethanolic extracts of *Perilla* leaves, but the flavonoids concentration did not significantly differ between the two types of extracts. Zhao et al. investigated the total polyphenols concentration from 44 species of *Perilla frutescens* using ultrasonic-assisted ethanol extraction (60%) and ultrasound-assisted cellulase hydrolysis, and demonstrated that the total phenols concentration was significantly higher when using cellulase hydrolysis extracts compared to ethanolic extracts [27].

The amount of caffeic acid in the ethanolic extracts of *Perilla frutescens* was substantially higher, with the greatest concentration found in the E1 extract. Indeed, it was found that the total concentration of caffeic acid varies among various strains of *Perilla frutescens*, although less than rosmarinic acid, and this might be due to different genetic backgrounds of the plant [28]. 

Additionally, it was discovered that ethanolic extracts had considerably greater levels of syringic acid, *p*-coumaric acid, ferulic acid, kaempferol, isorhamnetin, and pinocembrin. *P*-coumaric acid, isorhamnetin, and pinocembrin were most abundant in E1 extract, but syringic acid was most abundant in E2. Kaempferol and ferulic acid were abundant in the E3 extract. Other studies have confirmed the presence of these compounds in different varieties of *Perilla frutescens* [29,30,31]. 

Polyphenolic compounds exhibit antioxidative properties through various mechanisms. The hydroxyl groups serve as effective hydrogen donors and can engage with reactive oxygen and nitrogen species, as discussed by Valentao et al. [32] and Heim et al. [33]. This interaction leads to a termination reaction that effectively halts the generation of free radicals. Consequently, the initial reactive species transform into a radical form of the antioxidant, characterized by significantly enhanced chemical stability compared to the initial radical state. 

The antioxidative capacity of phenolic compounds is also associated with their capability to chelate metal ions involved in free radical production [34]. However, it is important to note that phenols can, under certain circumstances, act as pro-oxidants. This can occur through the chelation of metals that either maintain or enhance their catalytic activity or by reducing metals, subsequently increasing their propensity to generate free radicals [35].

Moreover, the structural attributes of phenolic compounds make them prone to interacting with proteins, owing to their hydrophobic benzenoid rings and the hydrogen-bonding potential of phenolic hydroxyl groups. Consequently, phenolics possess the capacity to act as antioxidants by inhibiting specific enzymes involved in radical generation, such as various cytochrome P450 isoforms, lipoxygenases, cyclooxygenase, and xanthine oxidase [36].

In this study, we demonstrated that the iron chelation capacity, the scavenging capacity of hydroxyl radical, the scavenging capacity of the superoxide anion radical, and the lipoxygenase inhibition capacity increased with *Perilla frutescens* extracts concentrations, and they were significantly higher in ethanolic extracts. 

These results could be explained by the fact that the retrieval of antioxidant compounds from botanical sources typically involves employing various extraction methodologies tailored to their inherent chemistry and non-uniform distribution within the plant matrix. Among these methods, solvent extraction stands out as the most commonly utilized technique for the isolation of antioxidant compounds from plants [37].

However, it is crucial to note that the yields of extracted substances, the polyphenolic contents, and the resultant antioxidant properties of plant materials are markedly influenced by the choice of extracting solvent and the extraction method. This variation arises due to the presence of diverse antioxidant compounds with distinct chemical properties and polarities, which may or may not be soluble in a given solvent [37].

In practice, polar solvents are frequently selected for the recovery of polyphenols from plant matrices. Of these, the most suitable options include aqueous mixtures, either hot or cold, incorporating ethanol, methanol, acetone, and ethyl acetate [37].

As far as we know, the cytotoxic effects of *Perilla frutescens* extracts on tumoral cell cultures have barely been studied. For example, alcoholic extracts (ethanol) from *Perilla frutescens* leaves were shown to have antitumoral effects in vitro, inhibiting the adhesion, proliferation and colony formation of human colon and lung tumor cells [38]. Moreover, it was proved that isoegomaketone extracted from *Perilla frutescens* can induce in vitro apoptosis in human melanoma cells [38] and in human breast tumor cells [39]. However, to our knowledge, this was the first time *Perilla frutescens* extracts were tested on human tumoral osteoblasts (MG-63 cell line) and human tumoral keratinocytes (A431 cell line).

The results indicated that in MG-63 cell line, the antitumoral effects were evident for all extracts at concentrations ≥ 0.5 mg/mL. In contrast, cytocompatibility with the MG-63 cell line was observed at concentrations of 0.2 mg/mL for all extracts. Notably, perillyl alcohol exhibited antitumoral effects at concentrations ≥ 0.5 mg/mL. In the case of the MG-63 cell line, extracts E1–E3 and A1–A3, with viability values ranging from 101% to 106%, demonstrated cell densities comparable to those in the control group. However, perillyl alcohol, with a viability of 82%, displayed areas devoid of cells.

Regarding the A431 cell line, cytocompatibility was observed at concentrations of 0.05 mg/mL for E1 and E3 extracts, 0.1 mg/mL for E2, A1, A2, and A3 extracts, and 0.2 mg/mL for perillyl alcohol. Moreover, extracts E3 and A1–A2, with viability values of approximately 98%, exhibited cell densities similar to the control group. Conversely, extract E1, resulting in a 72 h cell viability of 82%, displayed a reduced cell density compared to the control.

These results provide additional data for researchers who are studying the effects of herbal extracts on human tumoral osteoblasts and human tumoral keratinocytes. The cytotoxic concentrations of *Perilla frutescens* extracts need to be determined in various cell cultures, and our results showed that perillyl alcohol and various types of *Perilla frutescens* extract could serve as potential cytotoxic agents in the studied cell lines.

Data from other studies confirmed the cytotoxic effects of perillyl alcohol on H520 (non-small cell lung cancer) [40], A549 (human lung cancer), HepG2 (human liver cancer) cell lines [41], and BroTo (human tongue squamous cell carcinoma) [42] cell lines. On the other hand, data on the cytotoxic effects of extracts from *Perilla frutescens* varieties *a* are missing.

Further studies, on various type of oncogenic cell lines, and using different extracts of *Perilla frutescens* could confirm our results. Overall, all ethanolic extracts exhibited higher concentrations of phenols and antioxidant activity compared to mixed solvents extracts, thus supporting their use in pharmacological practice.

## 5. Conclusions

The total concentrations of polyphenols and anthocyanins were significantly higher in the ethanolic extracts of *Perilla frutescens*, while the concentration of flavonoids was not significantly different between the two types of extracts.

In this study, we demonstrated that with increasing concentrations of *Perilla frutescens* extracts, iron chelating capacity, hydroxyl radical scavenging capacity, superoxide anion radical scavenging capacity, and lipoxygenase inhibition capacity increased and were significantly higher for the ethanolic extracts.

The demonstrated cytotoxic effects of perillyl alcohol on MG-63 cell line and of E1 extract (*Perilla frutescens* var. *crispa* f. *purpurea*) on A431 cell line could serve as a basis for further cytotoxicity studies. 

## Figures and Tables

**Table 1:** The coding of the extracts and their extraction yield.

Solvent	*Perilla frutescens* Species Variety	Extract Code	Extraction Yield (%/Mean and Standard Deviation)
70% Ethanol	*Perilla frutescens* var. *crispa* f. *purpurea*	E1	58.22% (* 2.04: 1.18 ± 0.03) ^ab^
70% Ethanol	*Perilla frutescens* var. *frutescens* f. *viridis*	E2	58.79% (* 2.03: 1.19 ± 0.01) ^a^
70% Ethanol	*Perilla frutescens* var. *frutescens* f. *crispidiscolor*	E3	48.32% (* 2.02: 0.97 ± 0.01) ^c^
Acetone: Ethanol (7:3) + Citric Acid	*Perilla frutescens* var. *crispa* f. *purpurea*	A1	38.51% (* 2.01: 0.77 ± 0.006) ^e^
Acetone: Ethanol (7:3) + Citric Acid	*Perilla frutescens* var. *frutescens* f. *viridis*	A2	34.84% (* 2.04: 0.71 ± 0.02) ^f^
Acetone: Ethanol (7:3) + Citric Acid	*Perilla frutescens* var. *frutescens* f. *crispidiscolor*	A3	44.47% (* 2.02: 0.90 ± 0.01) ^d^

Table legend: * Dry mass. Means not sharing any letter are significantly different.

**Table 2:** Total phenols, flavonoids and anthocyanins quantification from ethanolic and ethanol–acetone extracts.

Sample	Total Phenols (mg Gallic Acid/mL Extract)	Total Flavonoids (mg Rutoside/mL Extract)	Anthocyanins (mg Cyanidol/mL Extract)
E1	2.15 ± 0.12 ^b^	0.17 ± 0.02 ^a^	0.10 ± 0.001 ^a^
E2	2.36 ± 0.11 ^a^	0.09 ± 0.002 ^c^	0.007 ± 0.007 ^c^
E3	2.13 ± 0.03 ^c^	0.11 ± 0.01 ^bc^	0.08 ± 0.001 ^b^
A1	0.33 ± 0.02 ^d^	0.11 ± 0.009 ^bcd^	0.09 ± 0.001 ^d^
A2	0.14 ± 0.03 ^f^	0.08 ± 0.002 ^cf^	-
A3	0.28 ± 0.02 ^de^	0.10 ± 0.001 ^bcdef^	0.06 ± 0.001 ^e^

Legend: E—ethanolic extract; A—acetone: ethanol extracts. Means not sharing any letter are significantly different.

**Table 3:** Quantification of various compounds from *Perilla frutescens* extracts using UHPLC.

Compound	Identified Compound (µg/mL Extract)					
	E1	E2	E3	A1	A2	A3
Gallic Acid	0.249 ^a^	0.235 ^ab^	0.095 ^ac^	-	0.225 ^ad^	0.103 ^ae^
Catechin	1.526 ^c^	2.025 ^ac^	1.672 ^bc^	1.388 ^ec^	1.355 ^fc^	1.581 ^dc^
Chlorogenic Acid	1.15 ^bc^	0.388 ^cd^	4.448 ^a^	0.376 ^cedf^	0.633 ^ced^	0.331 ^cef^
Caffeic Acid	109.714 ^a^	55.12 ^c^	81.306 ^b^	8.582 ^e^	12.022 ^d^	7.108 ^f^
Epi-Catechin	1.014 ^abc^	0.870 ^abc^	0.912 ^abc^	0.769 ^abce^	0.653 ^bcef^	1.030 ^abcd^
Syringic acid	129.323 ^c^	244.033 ^a^	232.475 ^b^	20.57 ^e^	35.109 ^d^	19.603 ^f^
*P*-Coumaric Acid	11.163 ^a^	10.069 ^b^	7.097 ^c^	2.222 ^f^	2.25 ^fd^	3.788 ^d^
Ferulic Acid	2.423 ^abc^	2.429 ^abc^	2.551 ^abc^	-	0.538 ^e^	0.722 ^d^
Ellagic Acid	9.930 ^a^	5.865^b^	4.807 ^c^	3.421 ^e^	3.677 ^d^	2.731 ^f^
Cinnamic Acid	70.465 ^a^	-	22.698 ^b^	16.544 ^d^	11.494 ^e^	25.218 ^c^
Rosmarinic Acid	67.665 ^a^	10.351 ^c^	14.871 ^b^	1.098 ^f^	3.177 ^e^	9.004 ^d^
Quercitin	19.663 ^a^	3.912 ^c^	16.828 ^b^	-	-	12.243 ^d^
Kaemferol	36.45 ^bc^	38.12 ^bc^	41.09 ^a^	22.05 ^de^	26.15 ^de^	19.47 ^ef^
Isorhamnetin	1.194 ^a^	0.805 ^bc^	0.745 ^bc^	0.138 ^ef^	0.101 ^def^	0.263 ^de^
Apigenin	3.400 ^c^	8.361 ^b^	10.656 ^a^	2.677 ^e^	4.061 ^d^	1.313 ^f^
Pinostrobin	31.827 ^a^	28.35 ^b^	24.081 ^cd^	23.601 ^e^	-	24.320 ^cd^
Pinocembrin	2.323 ^ab^	0.972 ^c^	2.302 ^ab^	0.387 ^fd^	0.447 ^def^	0.424 ^def^
Crysin	1.11 ^ce^	4.214 ^a^	3.239 ^b^	1.449 ^de^	1.354 ^cde^	0.56 ^f^

Table legend: Letters indicate statistical significance according to the Compact Letter Display system. Means not sharing any letter are significantly different.

**Table 4:** Determination of the in vitro antioxidant action of *Perilla frutescens* extracts.

Samples	Iron Chelation Capacity Assessment—IC50 (μg/mL Final Solution)
E1	166.36 ± 0.18 ^c^
E2	386.69 ± 9.36 ^a^
E3	297.01 ± 0.31 ^b^
A1	811.88 ± 1.32 ^g^
A2	1040.93 ± 0.17 ^e^
A3	830.49 ± 0.82 ^f^
Gallic acid	1163.15 ± 2.63 ^d^
Samples	Determination of Hydroxyl Radical Scavenging Capacity—IC50 (μg/mL final solution)
E1	122.62 ± 0.74 ^c^
E2	154.58 ± 0.41 ^ab^
E3	153.16 ± 1.11 ^ab^
A1	156.96 ± 3.57 ^abe^
A2	-
A3	-
Gallic acid	177.29 ± 0.66 ^d^
Samples	Determination of Superoxide Anion Scavenging Capacity—IC50 (μg/mL final solution)
E1	321.60 ± 9.12 ^c^
E2	520.79 ± 16.90 ^ab^
E3	470.08 ± 7.32 ^ab^
A1	977.47 ± 39.37 ^e^
A2	-
A3	1741.66 ± 29.87 ^d^
Gallic acid	543.38 ± 15.43 ^af^
Samples	Determination of the lipoxygenase inhibition capacity—IC50 (μg/mL final solution)
E1	85.99 ± 1.85 ^c^
E2	134.77 ± 2.49 ^a^
E3	104.10 ± 5.05 ^b^
A1	-
A2	-
A3	-
Gallic acid	28.85 ± 0.76 ^d^

Table legend: IC50—Half maximal inhibitory concentration. Means that do not share any letter are significantly different.

**Table 5:** Intergroup comparison of MG-63 celullar viability considering the cytotoxic concentrations of *Perilla frutescens* extracts and Peryllyl alcohol.

Extracts	Time Frame	2 mg/mL	1 mg/mL	0.5 mg/mL	Controls
E1 (mean and standard deviation)	24 h	53.50 ± 1.32 ^abc^	48.98 ± 3.95 ^abc^	47.03 ± 3.67 ^abc^	100.00 ± 7.19 ^d^
E1 (mean and standard deviation)	48 h	47.33 ± 11.18 ^abc^	45.54 ± 3.82 ^abc^	41.33 ± 2.68 ^abc^	100 ± 9.16 ^d^
E1 (mean and standard deviation)	72 h	43.36 ± 1.94 ^abc^	40.99 ± 2.53 ^abc^	42.20 ± 1.99 ^abc^	100 ± 20.64 ^d^
E2 (mean and standard deviation)	24 h	48.74 ± 1.46 ^abc^	47.76 ± 2.60 ^abc^	49.84 ± 5.54 ^abc^	100.00 ± 7.19 ^d^
E2 (mean and standard deviation)	48 h	39.70 ± 3.20 ^abc^	43.05 ± 6.69^abc^	42.50 ± 5.33 ^abc^	100 ± 9.16 ^d^
E2 (mean and standard deviation)	72 h	40.24 ± 4.64 ^abc^	45.68 ± 4.15 ^abc^	38.12 ± 2.56 ^abc^	100 ± 20.64 ^d^
E3 (mean and standard deviation)	24 h	51.91 ± 2.11 ^a^	48.28 ± 3.12 ^a^	53.69 ± 4.21 ^a^	100.00 ± 7.19 ^d^
E3 (mean and standard deviation)	48 h	44.21 ± 5.9 ^bc^	41.26 ± 1.63 ^bc^	44.21 ± 6 ^bc^	100 ± 9.16 ^d^
E3 (mean and standard deviation)	72 h	43.64 ± 4.69 ^bc^	39.18 ± 3.43 ^bc^	45.99 ± 2.55 ^bc^	100 ± 20.64 ^d^
A1 (mean and standard deviation)	24 h	51.21 ± 8.12 ^a^	55.34 ± 6.88 ^a^	49.84 ± 8.37 ^a^	100.00 ± 7.19 ^d^
A1 (mean and standard deviation)	48 h	43.20 ± 3.86 ^bc^	42.35 ± 4.41 ^bc^	40.09 ± 2.30 ^bc^	100 ± 9.16 ^d^
A1 (mean and standard deviation)	72 h	39.40 ± 2.02 ^bc^	39.71 ± 2.10 ^bc^	43.72 ± 3.86 ^bc^	100 ± 20.64 ^d^
A2 (mean and standard deviation)	24 h	45.994 ± 2.95 ^abc^	47.46 ± 4.57 ^abc^	47.55 ± 3.21 ^abc^	100.00 ± 7.19 ^d^
A2 (mean and standard deviation)	48 h	38.22 ± 1.76 ^abc^	44.60 ± 5.56 ^abc^	38.82 ± 4.53 ^abc^	100 ± 9.16 ^d^
A2 (mean and standard deviation)	72 h	42.20 ± 3.18 ^abc^	41.30 ± 5.59 ^abc^	43.64 ± 6.53 ^abc^	100 ± 20.64 ^d^
A3 (mean and standard deviation)	24 h	45.17 ± 6.06 ^abc^	43.15 ± 3.32 ^abc^	47.27 ± 6.18 ^abc^	100.00 ± 7.19 ^d^
A3 (mean and standard deviation)	48 h	37.99 ± 4.90 ^abc^	36.97 ± 3.03 ^abc^	39.47 ± 2.56 ^abc^	100 ± 9.16 ^d^
A3 (mean and standard deviation)	72 h	43.57 ± 2.45 ^abc^	40.69 ± 2.4 ^abc^	44.402 ± 1.66 ^abc^	100 ± 20.64 ^d^
Perillyl alcohol (mean)	24 h	57.53 ± 5.82 ^a^	54.33 ± 9.36 ^a^	95.37 ± 7.03 ^a^	100.00 ± 7.19 ^d^
Perillyl alcohol (mean)	48 h	46.16 ± 2.23 ^bc^	43.98± 4.62 ^bc^	81.69 ± 10.88 ^b^	100 ± 9.16 ^d^
Perillyl alcohol (mean)	72 h	48.63 ± 2.43 ^bc^	45.68 ± 4.37 ^bc^	42.66 ± 2.15 ^c^	100 ± 20.64 ^d^

Table legend: Letters indicate statistical significance according to the Compact Letter Display system. Means not sharing any letter are significantly different.

**Table 6:** Intergroup comparison of A431 cellular viability considering the cytotoxic concentrations of *Perilla frutescens* extracts and perillyl alcohol.

Extracts	Time Frame	2 mg/mL	1 mg/mL	0.5 mg/mL	Controls
E1 (mean and standard deviation)	24 h	13.84 ± 0.40 ^abc^	13.76 ± 0.91 ^abc^	14.40 ± 0.56 ^abc^	100 ± 6.08 ^d^
E1 (mean and standard deviation)	48 h	10.29 ± 1.73 ^abc^	9.96 ± 0.89 ^abc^	11.29 ± 0.99 ^abc^	100 ± 9.58 ^d^
E1 (mean and standard deviation)	72 h	5.63 ± 0.23 ^abc^	5.46 ± 0.65 ^abc^	5.02 ± 0.53 ^abc^	100 ± 8.91 ^d^
E2 (mean and standard deviation)	24 h	14.57 ± 1.38 ^abc^	15.94 ± 0.63 ^abc^	16.54 ± 2.98 ^abc^	100 ± 6.08 ^d^
E2 (mean and standard deviation)	48 h	10.66 ± 2.2 ^abc^	9.94 ± 1.15 ^abc^	10.17 ± 0.68 ^abc^	100 ± 9.58 ^d^
E2 (mean and standard deviation)	72 h	4.73 ± 0.38 ^abc^	5.183 ± 0.44 ^abc^	5.13 ± 0.74 ^abc^	100 ± 8.91 ^d^
E3 (mean and standard deviation)	24 h	15.65 ± 1.57 ^abc^	13.89 ± 1.46 ^abc^	15.17 ± 1.21 ^abc^	100 ± 6.08 ^d^
E3 (mean and standard deviation)	48 h	9.92 ± 0.60 ^abc^	9.99 ± 0.65 ^abc^	10.15 ± 1.69 ^abc^	100 ± 9.58 ^d^
E3 (mean and standard deviation)	72 h	5.21 ± 0.63 ^abc^	4.88 ± 0.52 ^abc^	5.66 ± 0.09 ^abc^	100 ± 8.91 ^d^
A1 (mean and standard deviation)	24 h	14.63 ± 1.38 ^abc^	14.03 ± 0.43 ^abc^	13.60 ± 0.90 ^abc^	100 ± 6.08 ^d^
A1 (mean and standard deviation)	48 h	9.98 ± 1.26 ^abc^	10.93 ± 1.23 ^abc^	10.17 ± 0.89 ^abc^	100 ± 9.58 ^d^
A1 (mean and standard deviation)	72 h	5.24 ± 1.26 ^abc^	4.95 ± 1.23 ^abc^	4.89 ± 0.89 ^abc^	100 ± 8.91 ^d^
A2 (mean and standard deviation)	24 h	12.95 ± 1.07 ^a^	12.83 ± 0.77 ^a^	14.40 ± 0.72 ^a^	100 ± 6.08 ^d^
A2 (mean and standard deviation)	48 h	8.71 ± 0.47 ^b^	11.10 ± 1.98 ^b^	9.78 ± 0.64 ^b^	100 ± 9.58 ^d^
A2 (mean and standard deviation)	72 h	4.81 ± 0.44 ^c^	4.911 ± 0.75 ^c^	4.73 ± 0.52 ^c^	100 ± 8.91 ^d^
A3 (mean and standard deviation)	24 h	15.37 ± 0.56 ^a^	16.17 ± 0.43 ^a^	28.90 ± 2.15 ^a^	100 ± 6.08 ^d^
A3 (mean and standard deviation)	48 h	9.46 ± 0.80 ^b^	10.05 ± 1.65 ^b^	9.52 ± 1.05 ^b^	100 ± 9.58 ^d^
A3 (mean and standard deviation)	72 h	4.82 ± 0.46 ^c^	4.68 ± 0.29 ^c^	5.51 ± 0.41 ^c^	100 ± 8.91 ^d^
Perillyl alcohol (mean and standard deviation)	24 h	15 ± 0.97 ^ab^	13.88 ± 0.82 ^ab^	13.43 ± 0.86 ^ab^	100 ± 6.08 ^d^
Perillyl alcohol (mean and standard deviation)	48 h	11.50 ± 1.23 ^ab^	10.20 ± 1.64 ^ab^	17.78 ± 2.49 ^ab^	100 ± 9.58 ^d^
Perillyl alcohol (mean and standard deviation)	72 h	5.39 ± 0.35 ^c^	5.42 ± 0.25 ^c^	8.76 ± 2.05 ^c^	100 ± 8.91 ^d^

Table legend: Letters indicate statistical significance according to the Compact Letter Display system. Means not sharing any letter are significantly different.

## Data Availability

The data presented in this study are available on request from the corresponding author. The data are not publicly available due to local policies.

## References

[1] Adam G., Robu S., Flutur M.M., Cioanca O., Vasilache I.A., Adam A.M., Mircea C., Nechita A., Harabor V., Harabor A. (2023). Applications of *Perilla frutescens* Extracts in Clinical Practice. Antioxidants.

[2] Adam G., Adam A.M., Robu S., Harabor V., Harabor A., Nechita A., Marin D.B., Morariu I.D., Cioanca O., Vasilache I.A. (2023). The Effects of *Perilla frutescens* Extracts on IgA Nephropathy: A Systematic Review and Meta-Analysis. Pharmaceuticals.

[3] Huang S., Nan Y., Chen G., Ning N., Du Y., Lu D., Yang Y., Meng F., Yuan L. (2023). The Role and Mechanism of *Perilla frutescens* in Cancer Treatment. Molecules.

[4] Kim C.L., Shin Y.S., Choi S.H., Oh S., Kim K., Jeong H.S., Mo J.S. (2021). Extracts of *Perilla frutescens* var. Acuta (Odash.) Kudo Leaves Have Antitumor Effects on Breast Cancer Cells by Suppressing YAP Activity. Evid. Based Complement. Altern. Med..

[5] Zhou Y., Zheng J., Li Y., Xu D.P., Li S., Chen Y.M., Li H.B. (2016). Natural Polyphenols for Prevention and Treatment of Cancer. Nutrients.

[6] Ullah A., Munir S., Badshah S.L., Khan N., Ghani L., Poulson B.G., Emwas A.H., Jaremko M. (2020). Important Flavonoids and Their Role as a Therapeutic Agent. Molecules.

[7] Garcia P.J.B., Huang S.K., De Castro-Cruz K.A., Leron R.B., Tsai P.W. (2023). An In Vitro Evaluation and Network Pharmacology Analysis of Prospective Anti-Prostate Cancer Activity from *Perilla frutescens*. Plants.

[8] Meng L., Lozano Y.F., Gaydou E.M., Li B. (2008). Antioxidant activities of polyphenols extracted from *Perilla frutescens* varieties. Molecules.

[9] Lee J.E., Kim N., Yeo J.Y., Seo D.G., Kim S., Lee J.S., Hwang K.W., Park S.Y. (2019). Anti-Amyloidogenic Effects of Asarone Derivatives from *Perilla frutescens* Leaves against Beta-Amyloid Aggregation and Nitric Oxide Production. Molecules.

[10] Lee Y.H., Kim B., Kim S., Kim M.S., Kim H., Hwang S.R., Kim K., Lee J.H. (2017). Characterization of metabolite profiles from the leaves of green perilla (*Perilla frutescens*) by ultra high performance liquid chromatography coupled with electrospray ionization quadrupole time-of-flight mass spectrometry and screening for their antioxidant properties. J. Food Drug Anal..

[11] Guzik T.J., Korbut R., Adamek-Guzik T. (2003). Nitric oxide and superoxide in inflammation and immune regulation. J. Physiol. Pharmacol..

[12] Batir Marin D., Cioanca O., Apostu M., Tuchilus C., Mircea C., Robu S., Tutunaru D., Corciova A., Hancianu M. (2019). The Comparative Study of *Equisetum pratense*, *E. sylvaticum*, *E. telmateia*: Accumulation of Silicon, Antioxidant and Antimicrobial Screening. Rev. De Chim..

[13] Aron N., Bogdan-Goroftei R.-E., Boev M., Marin D., Ramos-Villarroel A., Iancu A.-V. (2023). Innovative Fermented Soy Drink with the Sea Buckthorn Syrup and the Probiotics Co-Culture of *Lactobacillus Paracasei* ssp. Paracasei (L. Casei^®^ 431) and *Bifidobacterium Animalis* ssp. Lactis (Bb-12^®^). Fermentation.

[14] Russo D. (2018). Flavonoids and the structure-antioxidant activity relationship. J. Pharmacogn. Nat. Prod..

[15] Amić D., Davidović-Amić D., Bešlo D., Trinajstić N. (2003). Structure-radical scavenging activity relationships of flavonoids. Croat. Chem. Acta.

[16] Pietta P.-G. (2000). Flavonoids as antioxidants. J. Nat. Prod..

[17] Lu W., Shi Y., Wang R., Su D., Tang M., Liu Y., Li Z. (2021). Antioxidant activity and healthy benefits of natural pigments in fruits: A review. Int. J. Mol. Sci..

[18] Mandal S., Hazra B., Sarkar R., Biswas S., Mandal N. (2011). Assessment of the antioxidant and reactive oxygen species scavenging activity of methanolic extract of *Caesalpinia crista* leaf. Evid.-Based Complement. Altern. Med..

[19] Humulescu I., Flutur M.-M., Cioanca O., Mircea C., Robu S., Marin-Batir D., Spac A., Corciova A., Hancianu M. (2021). Comparative chemical and biological activity of selective herbal extracts. Farmacia.

[20] Lungu I.I., Marin-Batîr D., Panainte A., Mircea C., Tuchiluș C., Ștefanache A., Szasz F.A., Grigorie D., Robu S., Cioancă O. (2023). Catechin-Zinc-Complex: Synthesis, Characterization and Biological Activity Assessment. Farmacia.

[21] Dinis T.C., Maderia V.M., Almeida L.M. (1994). Action of phenolic derivatives (acetaminophen, salicylate, and 5-aminosalicylate) as inhibitors of membrane lipid peroxidation and as peroxyl radical scavengers. Arch. Biochem. Biophys..

[22] Jeong Y., Lim D.W., Choi J. (2014). Assessment of Size-Dependent Antimicrobial and Cytotoxic Properties of Silver Nanoparticles. Adv. Mater. Sci. Eng..

[23] Wang Z., Luo D. (2007). Antioxidant activities of different fractions of polysaccharide purified from *Gynostemma pentaphyllum* Makino. Carbohydr. Polym..

[24] Malterud K.E., Rydland K.M. (2000). Inhibitors of 15-lipoxygenase from orange peel. J. Agric. Food Chem..

[25] Shang X., Zhang M., Hu J., Zhang Y., Yang L., Hou X. (2023). Chemical Compositions, Extraction Optimizations, and In Vitro Bioactivities of Flavonoids from Perilla Leaves (*Perillae folium*) by Microwave-Assisted Natural Deep Eutectic Solvents. Antioxidants.

[26] Chen M.L., Wu C.H., Hung L.S., Lin B.F. (2015). Ethanol Extract of *Perilla frutescens* Suppresses Allergen-Specific Th2 Responses and Alleviates Airway Inflammation and Hyperreactivity in Ovalbumin-Sensitized Murine Model of Asthma. Evid. Based Complement. Altern. Med..

[27] Zhao Y., Li H., Zhang Z., Ren Z., Yang F. (2022). Extraction, preparative monomer separation and antibacterial activity of total polyphenols from *Perilla frutescens*. Food Funct..

[28] Deguchi Y., Ito M. (2020). Caffeic acid and rosmarinic acid contents in genus Perilla. J. Nat. Med..

[29] Fujiwara Y., Kono M., Ito A., Ito M. (2018). Anthocyanins in perilla plants and dried leaves. Phytochemistry.

[30] Li Y., Zhang Y., Wang Y., Li X., Zhou L., Yang J., Guo L. (2022). Metabolites and chemometric study of Perilla (*Perilla frutescens*) from different varieties and geographical origins. J. Food Sci..

[31] Peng Y., Ye J., Kong J. (2005). Determination of phenolic compounds in *Perilla frutescens* L. by capillary electrophoresis with electrochemical detection. J. Agric. Food Chem..

[32] Valentão P., Fernandes E., Carvalho F., Andrade P.B., Seabra R.M., Bastos M.L. (2003). Hydroxyl radical and hypochlorous acid scavenging activity of small centaury (*Centaurium erythraea*) infusion. A comparative study with green tea (*Camellia sinensis*). Phytomedicine.

[33] Heim K.E., Tagliaferro A.R., Bobilya D.J. (2002). Flavonoid antioxidants: Chemistry, metabolism and structure-activity relationships. J. Nutr. Biochem..

[34] Yang C.S., Landau J.M., Huang M.T., Newmark H.L. (2001). Inhibition of carcinogenesis by dietary polyphenolic compounds. Annu. Rev. Nutr..

[35] Croft K.D. (1998). The chemistry and biological effects of flavonoids and phenolic acids. Ann. N. Y. Acad. Sci..

[36] Parr A., Bolwell G.P. (2000). Phenols in the plant and in man. The potential for possible nutritional enhancement of the diet by modifying the phenols content or profile. J. Sci. Food Agric..

[37] Sultana B., Anwar F., Ashraf M. (2009). Effect of extraction solvent/technique on the antioxidant activity of selected medicinal plant extracts. Molecules.

[38] Kwon S.J., Lee J.H., Moon K.D., Jeong I.Y., Ahn D.U., Lee M.K., Seo K.I. (2014). Induction of apoptosis by isoegomaketone from *Perilla frutescens* L. in B16 melanoma cells is mediated through ROS generation and mitochondrial-dependent, -independent pathway. Food Chem. Toxicol..

[39] Cho B.O., Jin C.H., Park Y.D., Ryu H.W., Byun M.W., Seo K.I., Jeong I.Y. (2011). Isoegomaketone induces apoptosis through caspase-dependent and caspase-independent pathways in human DLD1 cells. Biosci. Biotechnol. Biochem..

[40] Yeruva L., Pierre K.J., Elegbede A., Wang R.C., Carper S.W. (2007). Perillyl alcohol and perillic acid induced cell cycle arrest and apoptosis in non small cell lung cancer cells. Cancer Lett..

[41] Oturanel C.E., Kıran İ., Özşen Ö., Çiftçi G.A., Atlı Ö. (2017). Cytotoxic, Antiproliferative and Apoptotic Effects of Perillyl Alcohol and Its Biotransformation Metabolite on A549 and HepG2 Cancer Cell Lines. Anticancer Agents Med. Chem..

[42] Elegbede J.A., Flores R., Wang R.C. (2003). Perillyl alcohol and perillaldehyde induced cell cycle arrest and cell death in BroTo and A549 cells cultured in vitro. Life Sci..

